# Organic-Mineral Interaction between Biomimetic Materials and Hard Dental Tissues

**DOI:** 10.17691/stm2020.12.1.05

**Published:** 2020

**Authors:** P.V. Seredin, O.A. Uspenskaya, D.L. Goloshchapov, I.Yu. Ippolitov, Jitraporn (Pimm) Vongsvivut, Yu.A. Ippolitov

**Affiliations:** Senior Researcher, Department of Solid State Physics and Nanostructures, Voronezh State University, 1 University Square, Voronezh, 394018, Russia; Associate Professor, Head of the Department of Therapeutic Dentistry, Privolzhsky Research Medical University, 10/1 Minin and Pozharsky Square, Nizhny Novgorod, 603005, Russia; Leading Engineer, Department of Solid State Physics and Nanostructures, Voronezh State University, 1 University Square, Voronezh, 394018, Russia; Tutor, Department of Pediatric Dentistry and Orthodontics, Voronezh State Medical University named after N.N. Burdenko, 10 Studencheskaya St., Voronezh, 394036, Russia; Beamline Scientist, IR Microspectroscopy, The Australian Synchrotron (Synchrotron Light Source Australia Pty LTD), 800 Blackburn Rd., Clayton VIC 3168, Melbourne, Australia; Professor, Head of the Department of Pediatric Dentistry and Orthodontics, Voronezh State Medical University named after N.N. Burdenko, 10 Studencheskaya St., Voronezh, 394036, Russia

**Keywords:** biomimetic materials, native human hard dental tissue, IR microspectroscopy, synchrotron radiation

## Abstract

**The aim of the investigation** was to study the integration between native human dental tissue and new-generation biomimetic materials replicating the mineral-organic complex of dentin and enamel using IR microspectroscopy for multidimensional visualization and analysis.

**Materials and Methods:**

The conditions for stable integration at the interface between biomimetic material and natural hard tissue were identified using a biocomposite buffer system of nanocrystalline carbonate-substituted calcium hydroxyapatite corresponding in its total characteristics to human dentin-enamel apatite and a number of amino acids present in the organic matrix of dentin and enamel: L-histidine, L-lysine hydrochloride, L-arginine hydrochloride, and hyaluronic acid. The finished samples were studied using IR microspectroscopy on IRM channel equipment (The Australian Synchrotron, Melbourne, Australia).

**Results:**

The characteristic features of the biomimetic buffer layer at the interface between the enamel and dental material were revealed and visualized based on IR mapping of absorption intensity for particular functional molecular groups with the use of synchrotron radiation, location of the functional groups involved in the processes of biomimetic composite integration was identified.

## Introduction

Despite the recent advances in dental materials science, the cements and filling materials employed to restore the anatomic bases of human teeth or their parts have low affinity for the teeth enamel and dentin [1, 2]. As a result, there is poor adhesion and secondary caries most likely to occur at the boundary of dental filling materials and the enamel [2]. In this regard, improving the integration of applied or developed materials with the dental matrix is a challenge of modern dentistry [1, 3].

At the same time, numerous studies focus on the interaction of synthetic material with dental tissues as well as formation of a biomimetic buffer layer at the interface between the natural hard dental tissue and the dental composite [2–5] supposed to act as a link between two heterogeneous materials.

Given that dentin and enamel are structurally organized biological nanocrystalline composites with meso- and nanoporous structure, with anisotropy of mechanical, optical, and trophic properties [6], development of synthetic dental material with similar configuration is an extraordinary and very complicated problem [7]. Therefore, to minimize crown chipping, abrasion, erosion and caries activity at the boundary between biocomposite and dental tissue, a biomimetic approach to restoration and regeneration of lost hard dental tissues is actively developed today [8–10]. According to this approach, hard dental tissue restoration and reconstruction should be carried out with the use of materials closely resembling the apatite and amino acid matrix of natural enamel/dentin in their molecular composition, chemical and morphological properties [8, 10–12]. Modern dental materials mimicking native human dental tissue composition inevitably contain nanocrystalline calcium hydroxyapatite with various defective structures [7, 13, 14]. Besides, it is essential that such materials should contain various organic components to improve their mechanical, adhesive and strength properties [15–17].

It is important to note, there have been repeated attempts to use the biomimetic principle for reproducing the organic-mineral complex of teeth and achieving enamel- and dentin-like structures in composites [10, 18–21]. In the latest known research works, biocomposites have been created by synthesizing calcium hydroxyapatite in the presence of various polymers and amino acid components, as well as surfactants and high molecular weight compounds [15, 22–24]. This idea is based on the fundamental principles of material interaction and serves to achieve morphological homogeneity and homogeneous distribution of hydroxyapatite nanocrystals on the surface of polymer and organic matrix. However, the problem of synthesizing the biomimetic materials similar to enamel/dentin involves not only fundamental issues concerning biocomposite production technology [12, 25, 26], but also the tasks of establishing the organic-mineral interaction between the natural tissue and biomaterial replicating it [12, 27, 28]. Therefore, it is necessary not only to carry out thorough investigations of both biogenic samples of enamel/dentin and processes occurring in the synthetic analogues of native materials, but also to study interactions at the interface between dental material, biomimetic composite and hard tissues of the human tooth.

One of the methods that have proven effective in the study of biological objects is infrared (IR) Fourier-transform microspectroscopy [29–32]. The advantages of this method are its high selectivity and sensitivity: it offers the possibility to obtain extensive and diverse information on the molecular composition of various tissues of human teeth [30, 33]; to analyze the mechanisms of molecular transformations occurring in biomimetic materials; to reveal newly formed mineral phases [34, 35]. The advantages of IR microspectroscopy also involve the possibility to study multicomponent dental materials [30]. In contrast to some other methods, external influences on the system studied with IR microspectroscopy are weak; therefore, the obtained information does not undergo changes resulting from these interactions [31]. The use of a microscope in the measuring system and a synchrotron radiation source to study biological objects allows collecting large arrays of spectra from the sample microdomain in a short time. This makes it possible to form an IR microspectroscopic mosaic image of the sample, full of diverse information about molecular chemistry, composition, and structure of the studied heterogeneous samples simultaneously.

Application of synchrotron IR microspectroscopy has already enabled us to achieve the necessary spectral resolution and reliably identify changes that occur in the molecular composition of samples in case of caries [32, 36].

**The aim of the investigation** was to study molecular and chemical properties of the layer formed at the interface between the dental material, the biomimetic composite and the hard dental tissue using multidimensional visualization of synchrotron IR microspectroscopy data.

## Materials and Methods

Buffer layers of biocomposite materials were studied and conditions for achieving stable functional bonds at the interface between the biomimetic material and the natural hard tissue were identified using the specimens of teeth extracted from patients aged 18–45 years for orthodontic indications. To avoid long-term calcification processes, teeth specimens with initial fissure caries were prepared immediately in accordance with the diagnosed pathology and approved standards.

In all specimens, a cavity was formed in the enamel preserving the untreated dentin, using a water-cooling system to avoid overheating of the dental matrix. The surface of the obtained cavity was covered with a biocomposite buffer system containing nanocrystalline carbonate-substituted calcium hydroxyapatite (CHA) developed by us and corresponding in the main characteristics to human dentin-enamel apatite [37, 38] and a number of amino acids present in the organic matrix of dentin/enamel: L-histidine, L-lysine hydrochloride, L-arginine hydrochloride, and hyaluronic acid. The ratio of the components was chosen according to their content in the enamel matrix [39]. When fixing the obtained buffer system, a universal light-curing adhesive showing effective bonding to the developed commercial materials was used for the bioactive bonding system [40]. CHA was added to the adhesive to fill the defects and improve bonding to the hard dental tissues. The adhesive containing CHA was applied to the surface of the buffer layer and preliminarily photopolymerized for 20 s. Within 1 min, the Dyract XP commercial restorative dental compomer (Dentsply Sirona, USA) containing adhesive components was applied onto the formed biocomposite bond layer. Finally, plane-parallel sections of the restored teeth specimens were prepared using the technique described in papers [41, 42].

**Figure 1:**
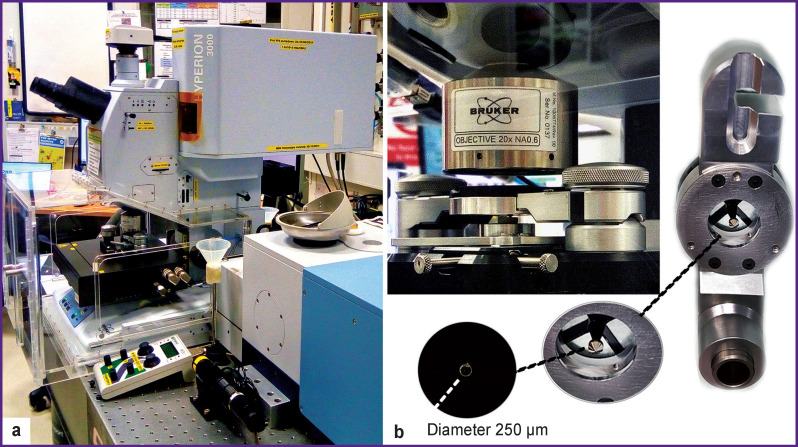
Hyperion 3000 IR microscope (a) and the attenuated total reflection prism for macro studies (Hybrid macro ATR-FTIR) (b)

The resulting slices were studied by IR microspectroscopy using optical channel equipment for IR microspectroscopy (The Australian Synchrotron; ASP, Melbourne, Australia) coupled with Vertex 80v IR spectrometer and Hyperion 3000 IR microscope with a detector cooled by liquid nitrogen (Bruker Optik, Germany) [31]. The selected areas of the studied biological objects were mapped using Hyperion 3000 IR microscope (Figure 1 (a)) equipped with the Hybrid macro ATR-FTIR prism and attenuated total internal reflection attachment (Figure 1 (b)). The size of the studied area was limited by the prism size and was ~250 μm. The obtained IR absorption spectra were recorded in the region of 3800–700 cm^–1^ at a spectral resolution of 4 cm^–1^.

The Australian source of synchrotron radiation with the macro ATR-FTIR attachment was used to study the sections of the interface between the dental material, the biomimetic composite, and the enamel. There were constructed IR maps of IR luminescence intensity for a particular functional molecular group in the area of dental material and tooth enamel integration using the algorithms presented in paper [31].

**Figure 2:**
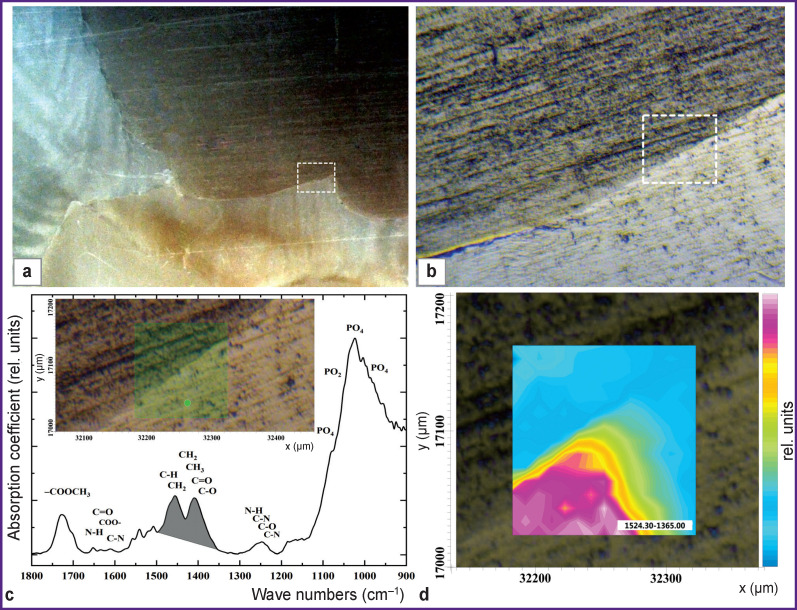
The image of a human tooth sample, ×5, (a) at the interfacial area the dental material, the biomimetic composite, and the enamel (b); typical IR absorption spectrum from the interface region (c); the map of total absorption (d)

Figure 2 shows an optical image of a plane-parallel section of the test sample whose enamel was restored using a biomimetic composite. The rectangular area denotes the interface between the dental material, the biomimetic composite, and the enamel. The visible part of the boundary region under consideration was determined by the size of the Hybrid macro ATR-FTIR prism window and was 250 μm, while the size of the studied region was 100×100 μm (Figure 2 (b)).

Studies of the interfacial boundary between the light-cured dental material, the biomimetic composite, and the enamel made it possible to determine a set of basic vibrational modes in the IR spectrum that can act as spectroscopic signatures of molecular groups corresponding to the materials present in the integration area. Figure 2 (c) shows the characteristic IR absorption spectrum obtained from the specified area on the sample surface. The main bands observed in the spectrum belong to the ester group (–COOCH_3_) present in the dental material composition based on Bis-GMA (1725 cm^–1^) [30], molecular groups CH_2_–CH_3_ (1457 cm^–1^), and amide bands at 1650 cm^–1^ (Amid I), 1550 cm^–1^ (Amid II), 1240 cm^–1^ (Amid III), present in both biomimetic composite and native tissue, they also belong to the inorganic components of enamel apatite and biocomposite (PO_4_ group at 1100–900 cm^–1^).

There is no morphological/molecular information on the presented IR map of complete absorption spectrum of the interfacial area between the dental material, biomimetic composite and the enamel of the sample. This IR map displays color-coding only for the main band intensities in the absorption spectra from various points on the sample surface, where certain chemical components are present.

## Results and Discussion

**Figure 3:**
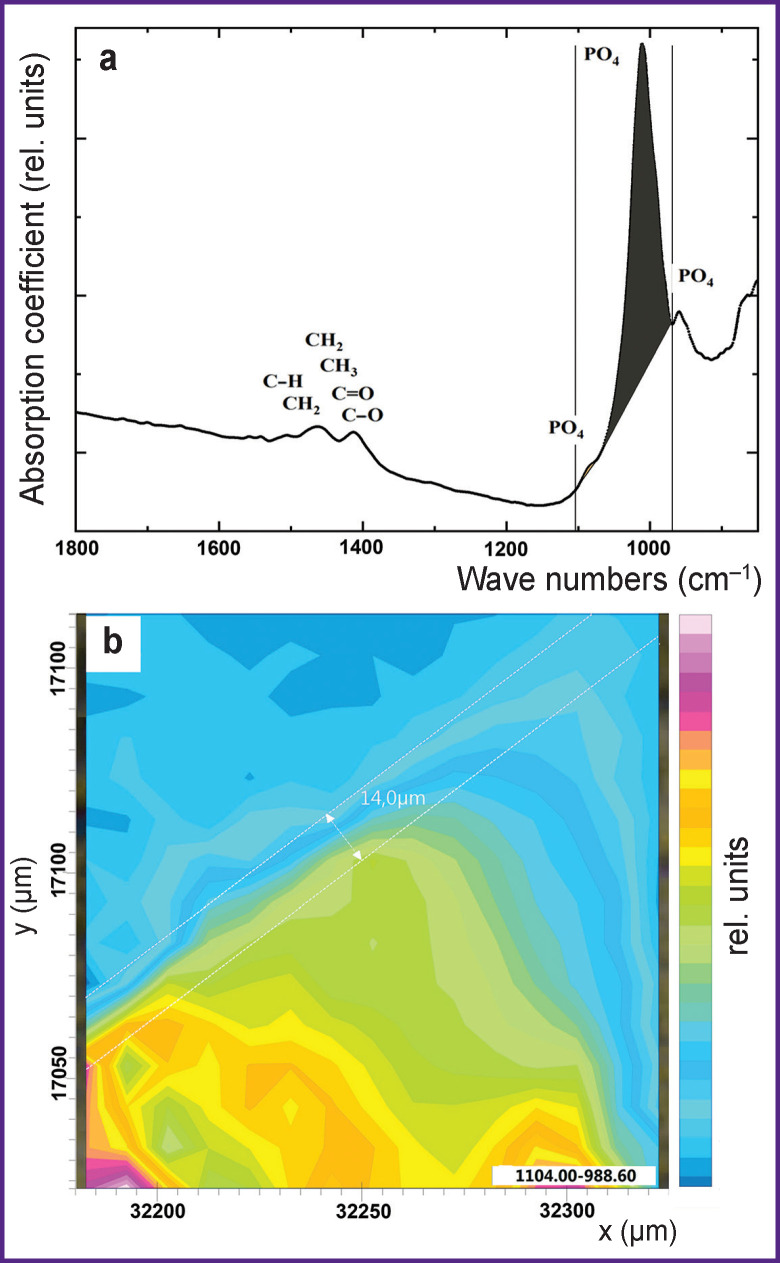
IR absorption spectrum from the enamel area of the sample, containing the characteristic phosphate mode in the range of 1104–988 cm^–1^, attributed to the enamel apatite (a); the characteristic IR image obtained through color-coding of absorption band intensity at 1104–988 cm^–1^ (b)

**Figure 4:**
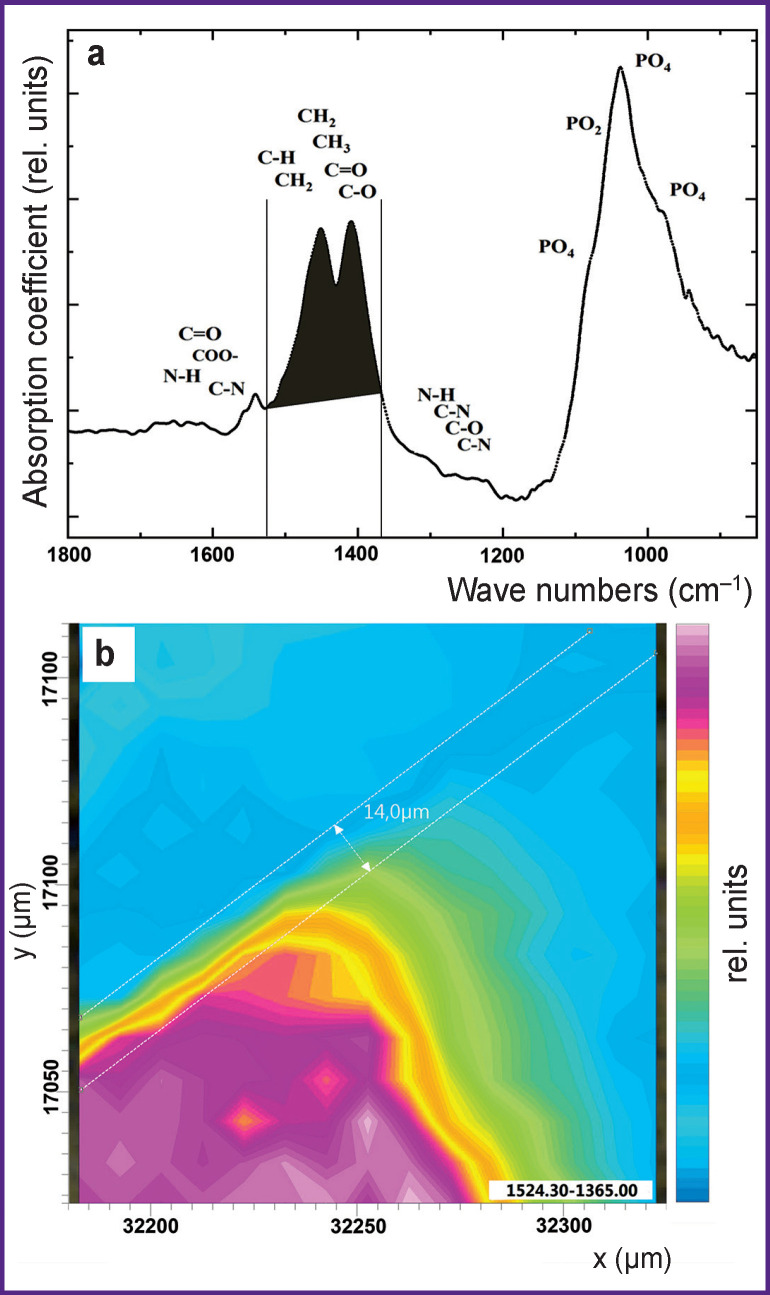
IR absorption spectrum of the sample with vibrational modes of organic component at 1524–1365 cm^–1^ (a); the characteristic IR image obtained through color-coding of this absorption band intensity (b)

**Figure 5:**
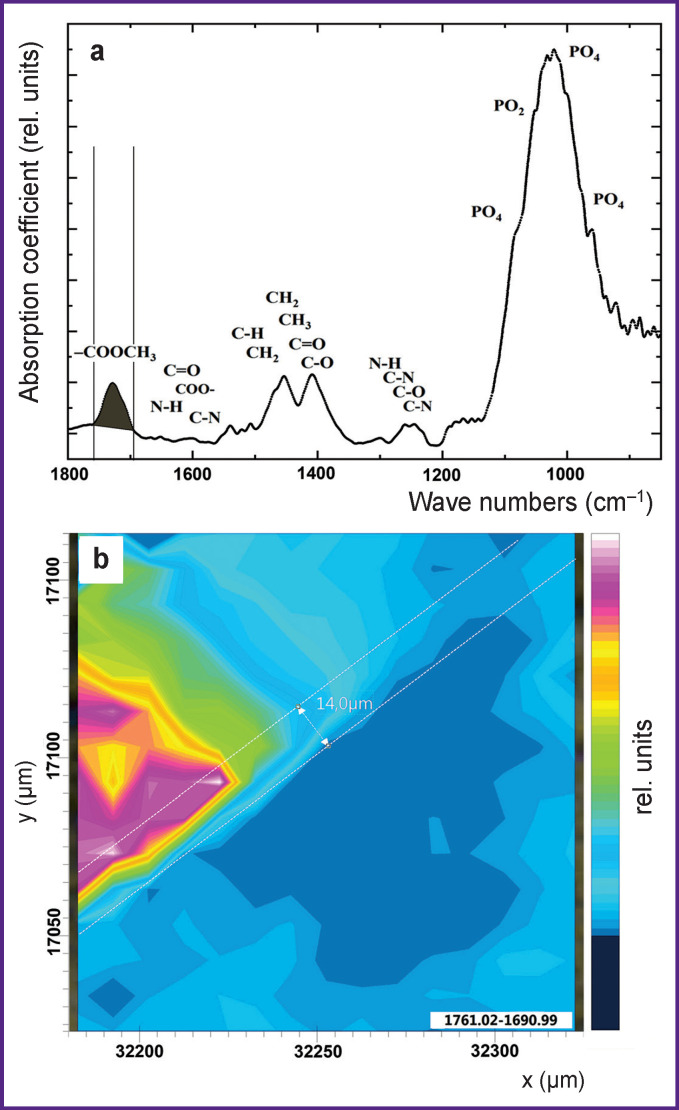
IR absorption spectrum with a characteristic feature in the region of 1761–1690 cm^–1^ belonging to the ester (–COOCH_3_) molecular group of the dental material (a); the characteristic IR image obtained through color-coding of this absorption band intensity (b)

The characteristic IR spectra obtained using synchrotron IR microspectroscopy from each of the studied areas in the interface between the dental material, the biomimetic composite and the enamel (Figures 3–5) contain spectroscopic signatures of molecular groups of materials present in the integration area.

The presented one-dimensional images (IR maps) produced based on color-coded intensities of three main spectral bands (1725, 1650–1240, and 1100–900 cm^–1^) contain information on the spatial distribution of the dental material, the organic and mineral (apatite) components across the studied sample section at the interfacial boundary of three media. Blue color encodes the lowest absorption intensity of a particular molecular group, while red indicates the highest one.

Figure 3 (a) shows the IR spectrum obtained from the enamel region of the sample (the lower right part of the biomimetic composite/tooth enamel integration area is in Figure 2 (b)). The frequency range of 1104–988 cm^–1^ highlighted in the spectrum belongs to the PO_4_ group of vibrations present in the composition of dental enamel apatite [30, 35, 36]. The obtained one-dimensional IR image (PO_4_ distribution in the sample segment) made it possible to detect visually the interfacial boundary between the enamel and the dental material. Information analysis shows that dental material area does not contain phosphate groups. The entire region adjacent to the enamel with active spectrum vibrations of nonzero intensity in the range 1104–988 cm^–1^ has dimensions of ~14 μm and is indicated in Figure 3 (b) with a broken line.

In the IR image of phosphate group distribution, the region with absorption intensity of PO_4_ group vibrations from 1.5 to 6.5 relative units is of particular interest (see Figure 3 (b)). This region is a biomimetic buffer layer whose composition (in this study) included CHA synthesized using our developed method. The IR spectra of this material were studied in paper [43]. Due to CHA contained in the biomimetic buffer layer, the dental material/enamel interface is clearly visible on the IR map where sharp color gradation is determined by vibration mode intensity of hydroxyapatite PO_4_ group.

It should be noted that IR image analysis showing only phosphate component distribution (see Figure 3 (b)) is not sufficient to study the processes of dental material integration with the tooth enamel mediated by the biomimetic buffer layer for the following reasons. Firstly, at the stage of sample preparation, both mechanical and chemical preparation of the dental tissue was performed to fix the buffer layer and the material (see “Materials and Methods”). Besides, IR absorption bands of phosphate groups can overlap with minor vibrations from the dental material in the region of 1100–900 cm**^–^**^1^, which makes the interphase boundary analysis ambiguous. Therefore, to obtain additional information about the interphase boundary area, there was constructed an IR image shown in Figure 4 (b). This IR map shows the distribution of absorption band intensity (CH_2_–CH_3_ groups and the amide component), correlating with molecular groups characteristic of the organic enamel component and present in the biomimetic buffer layer (Figure 4 (a)).

It is important to emphasize that the absorption band at the range of 1524–1365 cm**^–^**^1^ could relate to the characteristic vibrations from CHA of B-type [38]. However, as the results of our work [43] showed, vibrational modes of the organic enamel matrix component have a different characteristic profile and significantly higher intensity. Besides, the obtained IR spectrum of the dental enamel in paper [43] is completely similar to the experimental IR spectrum of the enamel in this work. This definitely characterizes the organic enamel component in the IR spectrum. It should be noted that along with the optical image of the analyzed sample area (see Figure 2 (a)), the constructed IR image for the group of bands at 1524–1365 cm**^–^**^1^ allows us to identify the region of the native dental enamel (see Figure 4 (b)).

Analysis of the IR image of the boundary area between the dental material, the biomimetic composite and the dental enamel (see Figure 4 (b)) clearly shows that the existing gradation of color-coded intensity of vibrational bands corresponding to the organic matrix is similar to that observed on the IR map of phosphate component distribution (see Figure 3 (b)). However, the distribution of the organic component is significantly more homogeneous in the buffer layer (see Figure 4 (b)) as compared to phosphate group distribution. This confirms the fact that the proportion of hydroxyapatite in the composition of the biomimetic buffer layer created by us at the stage of sample preparation is lower than that of the organic component.

Unfortunately, vibration range of 1524–1365 cm**^–^**^1^ (see Figure 4 (b)) selected by us for producing the IR image contains a number of overlapping bands in the IR spectrum, making it impossible to draw conclusions about the integration boundary forming between the enamel and dental light-cured cement as in the case with the IR map of inorganic component distribution.

To assess the interphase boundary more completely, the IR spectra of the Dyract XP commercial compomer restorative material has been studied. Analysis of the spectrum shown in Figure 5 (a) reveals that it contains a wide high-intensity vibrational mode in the range of 1100–1000 cm**^–^**^1^. This vibrational band may belong to the molecular group of aluminum silicate or silicon oxide used as light-cured cement fillers and is not associated with the phosphate components of the biomimetic material. At the same time, in the spectrum obtained from the dental material area, there is a group of vibrations in the range of 1600–1200 cm**^–^**^1^ because it contains synthetic additives applied for photopolymerization and bonding of dental cements based on Bis-GMA.

However, one more absorption band located at 1725 cm**^–^**^1^ can be seen in the IR spectrum from the dental material area (see Figure 5 (a)). As shown in paper [30], this band is a characteristic feature of the IR spectra of dental cements based on Bis-GMA and polymethylmethacrylate and belongs to the molecular group of the ester (–COOCH_3_). It is important to emphasize that this vibration does not overlap with other bands and, therefore, makes one-dimensional IR absorption analysis based on this parameter more reliable and informative as to the spatial distribution of dental material in the studied area.

It can be seen in Figure 5 (b) that the maximum intensity distribution of the vibrational mode of the ester group (–COOCH_3_) is similar to the location of the material observed in the optical image (see Figure 2 (b)). Particular attention should be paid to the area of dental material integration with the enamel where the drop in the given vibrational mode intensity — from maximum to minimum — is observed in the spatial range of ~14 μm and overlaps the region where the organic enamel component prevails (see Figure 4 (b)).

**Figure 6:**
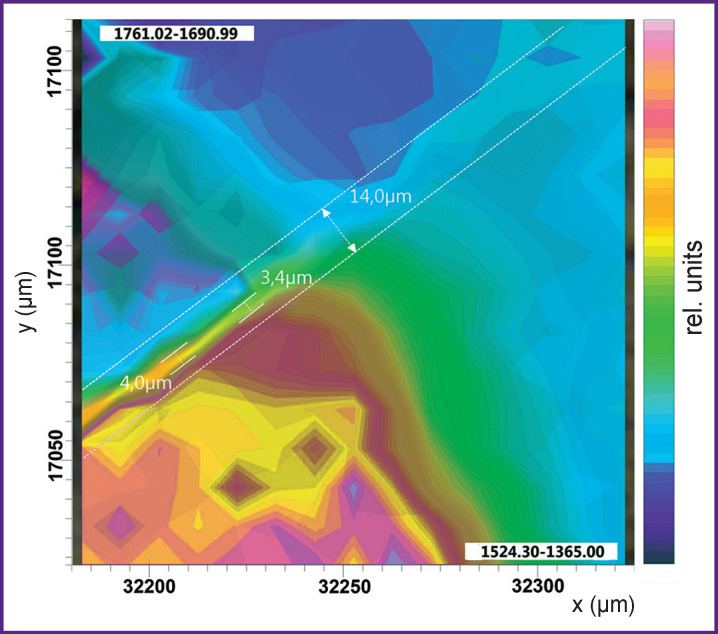
IR image obtained using the clustering method with account of the characteristic features of IR spectra in the regions of 1760–1690 and 1520–1360 cm^–1^

It is necessary to underline that simultaneous analysis of several IR maps based on the analysis of selected and even single bands does not always allow visualization of changes in the heterophase boundary between structurally similar materials. This is associated with the limitations of one-dimensional approach to detecting spectral changes. The above problems can be solved by using multidimensional clustering methods offering the possibility to classify vast arrays of component spectra efficiently. Using this approach, we managed to analyze the features of the complex interface between the dental material, the biomimetic composite, and the tooth enamel. Simultaneous analysis carried out with regard to all the features in the spectral ranges of 1760–1690 and 1520–1360 cm**^–^**^1^ revealed that the interaction between the dental material and the enamel occurred through a buffer layer. Figure 6 shows schematically the results of clustering, the biomimetic composite region marked.

It is schematically shown in the area marked with a broken line in Figure 6 that the buffer transition layer between the enamel and Dyract XP material is formed by bonding the enamel to partially demineralized enamel matrix, which suggests organic-mineral interaction taking place in the analyzed area. Based on the available data, it is reasonable to assume that the actual size of the buffer (integrating) layer is 3 to 4 μm. Thus, the data obtained by analyzing all IR images (see Figures 3–6) provide significant evidence on chemical differentiation of the functional groups of all materials in the boundary area between the biomimetic system and natural hard dental tissues, confirming the efficacy of the selected approach to analyzing the integration between dental cements and new-generation biomimetic composites.

## Conclusion

We have demonstrated the possibility of using molecular multidimensional IR imaging to study the integration between new-generation biomimetic materials replicating the mineral-organic complex of the enamel and native hard tissues of the human tooth.

Based on IR maps of intensity for particular functional molecular groups obtained using synchrotron radiation, we have revealed the differences between the healthy tissue, dental material and the biomimetic buffer layer at the interfacial areas and identified location and concentration of functional groups involved in the processes of biomimetic composite integration with native hard tissues of the human tooth.

The obtained microspectroscopy data reliably confirm the chemical differentiation of the materials and the presence of organic and mineral interaction at the interface between the biomimetic system and natural hard tissues of the human tooth.
